# Therapy of autoinflammatory diseases: results from an international registry

**DOI:** 10.1186/1546-0096-9-S1-P18

**Published:** 2011-09-14

**Authors:** NM ter Haar, J Frenkel, P Woo, L Cantarini, H Lachmann, A Insalaco, M Hofer, Y Uziel, S Ozen, S Nielsen, A Naselli, C Modesto, SM Al-Mayouf, I Kone-Paut, I Nikishina, N Iagaru, L Obici, E Papadopoulou-Alataki, D Rigante, C Boros, A Martini, N Ruperto, M Gattorno

**Affiliations:** 1 Department of Pediatrics University Medical Center Utrecht, Netherlands; 2 For the PRINTO and Eurofever Project

## Background

The evidence for therapy in autoinflammatory diseases is limited. There are few randomized controlled trials or disease registries.

## Aim

To evaluate the response to treatment of autoinflammatory diseases based on data of an international registry.

## Methods

A web-based registry collecting baseline and clinical information of autoinflammatory diseases was located at the member area of the PRINTO website (www.printo.it). Participating hospitals included pediatric rheumatology centers of the PRINTO network and adult centers with a specific interest in autoinflammatory diseases. Data was collected on Blau’s syndrome, Behçet’s disease, CAPS, CRMO, DIRA, FMF, MKD, NLRP12-mediated periodic fever, PAPA, PFAPA TRAPS and undefined periodic fevers. In total, 704 patients were included in this retrospective study on therapy.

## Results

NSAIDs and steroids were beneficial in most diseases. Anakinra induced a complete response in 70% of 40 treated CAPS patients, 82% of 28 TRAPS patients and in both DIRA patients. Furthermore, it was beneficial in 80% of 10 MKD patients. Etanercept was completely effective in all 6 Behçet patients, 34% of 32 TRAPS patients and 13% of 8 MKD patients and partially effective in 53% of TRAPS and 38% of MKD patients. Colchicine was beneficial in approximately 95% of 131 FMF and 18 Behçet patients, although the complete response rate was just 56% and 22%, respectively. For PFAPA syndrome, corticosteroids aborted the attacks in 78% of 113 patients.

## Conclusion

The results were compared to the literature. These combined findings could serve as a base for therapeutic guidelines and identify candidate drugs for future therapeutic trials.

